# Study of Hypoglycemic, Hypocholesterolemic and Antioxidant Activities of Iranian *Mentha Spicata *Leaves Aqueous Extract in Diabetic Rats

**Published:** 2017

**Authors:** Mahsan Bayani, Mahmood Ahmadi-hamedani, Ashkan Jebelli Javan

**Affiliations:** a *Faculty of Veterinary Medicine, Semnan University, Semnan, Iran. *; b *Department of Clinical Sciences, Faculty of Veterinary Medicine, Semnan University, Semnan, Iran.*; c *Department of Food Hygiene, Faculty of Veterinary Medicine, Semnan University, Semnan, Iran*.

**Keywords:** Alloxan, hypoglycemic, hypocholesterolemic, malondialdehyde, *Mentha spicata*

## Abstract

This study was conducted to evaluate the hypoglycemic, hypocholesterolemic and antioxidant properties of* Mentha spicata* (Labiateae) leaves aqueous extract (MSLA) in alloxan-induced diabetic rats. In this study, hyperglycemia was induced in male rats by intraperitoneal injection of alloxan monohydrate (150 mg/kg). The aqueous extract of *M. spicata* was orally administered at a dose of 300 mg/kg body weight to diabetic rats for 21 days and the effects were compared with glibenclamid (2 mg/kg). Fasing blood sugar (FBS), body weight, lipid profile and serum malondialdehyde (MDA) were monitored at 0, 7, 14 and 21 days after induction of diabetes. Total phenol contents (TP) and reducing power (RP) were also evaluated. TP and RP of aqueous extract were 2.763 ± 0.39 mg Galic acid/gr and 0.026 ± 0.001 EC_50 _mg/mL, respectively. The LD_50 _of the extract was found to be ˃ 1500 mg/kg. The administration of *M. spicata* aqueous extract produced a significant reduction (*P˂*0.01) in FBS, total cholesterol, triglyceride, low density lipoprotein-cholesterol and MDA (101.83 ± 4.33, 95.66 ± 4.75, 89.83 ± 5.26, 26.20 ± 5.10 mg/dl and 1.53 ± 0.61 µmol/l, respectively) in diabetic rats. These effects were comparable with the effects of standard antidiabetic drug (glibenclamide). The results of the investigation indicated that *M. spicata* leaf aqueous extract possess hypoglycemic, hypocholesterolemic and antioxidant properties in diabetic rats. Therefore, this study suggest a promising use of it for treatment of diabetes.

## Introduction

Diabetes mellitus is a chronic metaolic disease characterized by hyperglycemia resulting from absolute or relative lack of insulin or insulin insensitivity (1). Hyperglycemia is known as the most important risk factor for the development of cardiovascular diseases including retinopathy, neuropathy, nephropathy, diabetic ketoacidosis and diabetic coma (2). Imbalance between the production of Reactive oxygen species (ROS) such as superoxide, hydrogen peroxide, hydroxide radicals, and antioxidant defense mechanisms play an important role in the pathogenesis of diabetes mellitus (3). More than 800 plants have been identified with potential anti-diabetes which has more effective, less side effects and lower toxicity than synthetic compounds (4). *Mentha spicata* also Known as spearmint belongs to the Labiateae (Lamiaceae) family. The genus *Mentha* includes 25 to 30 species such as spearmint, peppermint, wild mint, corn mint, curled mint, bergamot, American mint, Korean mint, etc. of which spearmint is the most common of them (5,6). It is a rhizomatous, perennial plant growing 30–100 cm tall, with square-shaped and hairy stems and foliage, and a wide-spreading fleshy underground rhizome. The leaves are 5–9 cm long and 1.5–3 cm broad, with a serrated margin. Flowers spearmint is pink or white slender spikes (7). In Iran, spearmint commonly known as pooneh and its freesh and dried leaves is widely used as a stomach pain-relieving agent, antispasmodic, digestive and carminative (8). The main compounds isolated from different extracts of spearmin includes flavonoids, terpenoids, and phenols. There are monoterpenoids such as carvone, limonene, menthone, menthol, pulegone, dihydrocarvone, and *s*-carveol in spearmint oil (6). Aqueous leaf extract of this plant is used to relieve hiccup, flatulence, giddiness and as remedy for inflammation, bronchitis, and also to control vomiting during pregnancy (9). Anti-inflammatory, haemostatic and pain relieving properties have been identified in two new monoterpenoids (spicatoside, A and B) of the plant (10). Rosmarinic acid in essential oil of spearmint was found to have antimicrobial, insecticidal, antispasmodic, antiplatelet, and antioxidant properties and is widely used for the neutraceutical and cosmetic industries (11). Furthermore, antibacterial effects of *M. spicata* essential oil were determined on Listeria monocytogenes in traditional Lighvan cheese (12). With regard to the medicinal importance of the plant and its extensive use in traditional medicine, the objective of the present work is hence to study *in-vivo* hypoglycemic, hypocholesterolemic, and antioxidant activities of aqueous extract of Iranian *M. spicata* leaves.

## Experimental

*Chemicals and reagents*

Alloxan monohydrate (Sigma-Aldrich, St.Louis, MO, USA) and glucose oxidaseperoxidase strip (Accue-check diagnostic kit, Roche, Germany) were used in this study. Serum total cholesterol (TC), triglyceride (TG), and high-density lipoprotein (HDL)–cholesterol were estimated by using diagnostic kits (Pars Azmun, Tehran, Iran). All other chemicals used in this study were analytical grade.

*Animals*

Healthy Wistar albino rats in the weight range of 200–220g, bred in the laboratory animal unit of the Faculty of Veterinary Medicine, Semnan University of Iran, were selected for the experiments. The Rats were housed in stainless steel cages, supplied with fresh water and standard pellet diet ad libitum. They were maintained under standard conditions (12 h light/dark cycle; 22±3°C; 45%-55% humidity). All experimental protocols were approved by the Animal Research Ethical Committee of Semnan University, Faculty of veterinary science, Semnan, Iran.

*Plant material*

Fresh leaves of *Mentha piperita *and* Mentha spicata* were collected in October-November 2013 from suburb, Shahmirzad city (Semnan, province, Iran) and authenticated by the botany department of the Natural Resources and Animal Science, Research Center of Semnan, Iran and a voucher specimen of the plant was deposited in the herbarium of the center. 

*Preparation of extracts*

Dried leaves of these plants were prepared in the dark, grounded in a grinder and stored at refrigerator (4 °C) until use. Water extract was prepared by means of a soxhlet. In this regard, fruits samples were extracted with distilled water in a soxhlet apparatus until extraction water became colorless (13). Both extracts were further filtered and evaporated to dryness in a vacuum dryer (Rotary evaporator, RE-52AA, China).

*Determination of total phenolic contents*

Determination of phenolic compounds was accomplished as suggested by Barros *et al*. (14). In order to estimate total phenolics, 1 mL of the aqueous extract (5mg/mL) was combined with 1 mL Folin-Ciocalteu’s phenol reagent (Merck, Germany). Later on 1 mL saturated sodium carbonate solution (Merck, Germany) was added to the mixture after 3 min and total volume of mixture was adjusted to 10 mL with distilled water. This reaction mixture was then kept in dark for 90 min and then absorbance was read at 725 nm. Standard curve was calculated using Gallic acid.

*Measurement of Reducing Power*

The reductive potential of leaves aqueous extracts was determined according to the method described by Duh (15). The different concentrations of the extracts were made (0.05-1.6 mg/mL) in 0.2 M phosphate buffer pH 6.6 containing 1% potassium ferrocyanid Merck, Germany). The mixture was incubated at 50 °C for 20 min. A portion (2.5 mL) of trichloroacetic acid (10% w/v) was added to the mixture that was then centrifuged at 3000 xg for 10 min. The upper layer was separated and mixed with 2.5 mL distilled water containing 0.5 mL of ferric chloride 1% (Merck, Germany). The absorbance of this mixture was measured at 700 nm. The intensity in absorbance showed the antioxidant activities of the extracts. The concentration of extract which can provide absorbance of 0.5 (EC_50_) was determined from the graph of absorbance against concentration of extract.

*Acute toxicity study of the aqueous extract of Mentha spicata*

The acute toxicity of *Mentha spicata* leaf aqueous extract was determined by using fasting rats (200-220 gr), according to the method described by Ashraf *et al*. (16). It was observed that the aqueous extract of *Mentha spicata* was safe up to the dose of 1500 mg/kg b. w. Hence, 1/5^th^ (300 mg/kg b. w) of this dose was selected according to OECD guidelines No. 423 (Acute Toxic Class Method) to determine the antidiabetic activity (4).

*Induction of diabetes*

Diabetes was induced in Wistar albino rats by a single intra peritoneal injection of 150 mg/kg of Alloxan. The rats were fasted for 16 h prior to the induction of diabetes. Developing of diabetes was confirmed by measuring the fasting blood sugar (FBS) levels of rats using ACCU-Check glucose meter 6 days after the administration of alloxan. Rats with FBS of ≥ 126 mg/dl were considered diabetic (17).

*Experimental design*

The rats were randomLy divided into four groups (six rats in each group) as given below. Normal saline, Glibenclamide and MSLA was administered orally by gavage for 21 days to groups 1, 2, 3 and 4.

- Group I: normal control rats, received normal saline (10 mL/kg)
- Group II: diabaetic control rats received normal saline (10 mL/kg)
- Group III: diabaetic rats received glibenclamide (2 mg/kg/day, p.o.), 6 days after alloxan administration
- Group IV: diabaetic rats received MSLA (300 mg/kg/day, p.o.), 6 days after alloxan administration

Blood glocuse levels and body weights of the rats were estimated at weekly intervals on the 0th, 7th, 14th and 21th day of treatment. On 21th day of the study, all the animals were anesthesized in a chloroform chamber and also blood samples were collected from the hearts for serum lipid profile and lipid peroxidation (MDA) estimations. 

*Statistical analysis*

All the values of FBS, body weight, lipid profile and serum MDA were represented as mean ± SEM. The statistical of differences among the groups was analyzed using one-way analysis of variance (ANOVA) followed by Tukey’s multiple tests and values of p˂0.05 statistically considered significant.

## Results

*Comparison of* *in vitro* *antioxidant activity* *between* *MPLA* *and MSLA*

*Total phenolic* *capacity* *and reducing power*

 The total phenolic capacity and reducing power of MPLA and MSLA are shown in Table 1. The results showed that the total phenolic capacity and reducing power of MSLA are significantly (p˂0.05) better than MPLA. Therefore, the leaf aqueous extract of *M. spicata* was selected to evaluate its effect on blood glocuse as well as lipid and malondialdehyde levels *in-vivo* in alloxan-induced diabetic rats.

**Table 1:** Reducing power and total phenolic content of *M. spicata* and *M. piperita*

**Total Phenolic content** **(mg GA/g) **	**Reducing Power** **(IC**_50_ ** value in mg/mL)**	**Sample**
2.763±0.39	0.026±0.001**	*M. spicata*
1.759±0.34	0.137±0.020	*M. piperita*

Data are means ± SD of triplicate analyses of two mentha species.

* *P*<0.05 and

** *P*<0.001 total phenol and reducing power of* M.spicata* were compared with *M*. piperita.

**Table 2:** Effect of glibenclamide and MSLA on the FBS of alloxan-induced diabetic rats treated for 21 d.

		Mean fasting blood glocuse levels ± SE				
Group	Treatment	Pre-diabetic	Day 0	Day 7	Day 14	Day 21
1.	Normal saline (10 mL/kg)	79.25 ± 2.86	79.50 ± 2.39	79.00 ± 2.73	78.00 ± 2.27	72.75 ± 4.02
2.	Diabetic control (150 mg/kg)	83.25 ± 4.17	134.50 ± 2.98#	134.50 ± 3.42#	136.00 ± 2.16#	139.75 ± 3.32#
3.	Glibenclamide (2 mg/kg)	80.16 ± 1.49	140.33 ± 1.33	123.16 ± 3.72	122.83 ± 2.83	113.83 ± 3.68
4.	MSLA (300 mg/kg)	82.00 ± 5.24	136.00 ± 3.31	127.66 ± 3.09	108.16 ± 5.47^a^	101.83 ± 4.33^a^

Data are mean ± SEM, n=24. One-way ANOVA+Tukey test against diabetic rats

# *P*<0.0001 Diabetic control rats were compared with normal control rats.

* *P *< 0.05,

** *P *< 0.001 and

*** *P *< 0.0001 Diabetic treated rats were compared with diabetic control rats.

a *P *< 0.05,

b *P*<0.01 and

c *P*<0.0001 Diabetic treated rats with MSLA were compared with Diabetic treated rats with glinenclamide on corresponding day.

**Table 3:** Effect of glibenclamide and MSLA on the lipid profile of alloxan-induced diabetic rats

Group	Treatment	Total cholesterol (mg/dl)	Teriglycerides (mg/dl)	LDL (mg/dl)	HDL (mg/dl)
1.	Normal saline (10 mL/kg)	63.75±5.54	50.00±6.89	47.90±4.43	48.00±2.87
2.	Diabetic control (150 mg/kg)	180.66±5.54#	155.00±15.56#	105.66±9.13#	44.00±7.37
3.	Glibenclamide (2 mg/kg)	75.5±2.23*	81.83±7.19*	17.46±3.97*	41.66±2.99
4.	MSLA (300 mg/kg)	95.66±4.75*^a^	89.83±5.26*	26.20±5.10*	51.50±3.58^a^

Data are mean ± SEM, n=24. One-way ANOVA+Tukey test against hypercholesterolemic rats

# *P*<0.0001 Diabetic control rats were compared with normal control rats.

* *P*<0.0001 Diabetic treated rats were compared with diabetic control rats.

a *P*<0.05 Diabetic treated rats with MSLA were compared with Diabetic treated rats with glinenclamide.

*Effect of MSLA on the blood glucose levels of alloxan-induced diabetic rats*

Table 2 shows the changes in blood glucose level in normal, diabetic and on treatment for diabetes rats with glibenclamide and MSLA. The blood glocuse level was significantly increased (p˂0.001) in diabetic control rats (Group II) in comparison with normal control rats (Group I). Blood glucose level in rats that orally received the aqueous extract of *Mentha spicata* (300 mg/kg) significantly reduced (p˂0.001) compared to diabetic control. The aqueous extract 300 mg decreased blood glocuse level from 136.00 ± 3.31 to 101.83 ± 4.33 mg/kg. Hypoglycaemic effect was quite evident from 14th day onwards. Glibenclamide also significantly reduced (p˂0.001) blood glucose level from 140 to 113 mg/kg in rats that daily received the standard drug. The hypoglycemic effect of the aqueous extract of *Mentha spicata* was comparable and significantly (p˂0.05) more than glibenclamid.

*Effect of MSLA on body weight of alloxan-induced diabetic rats*

**Figure 1:**
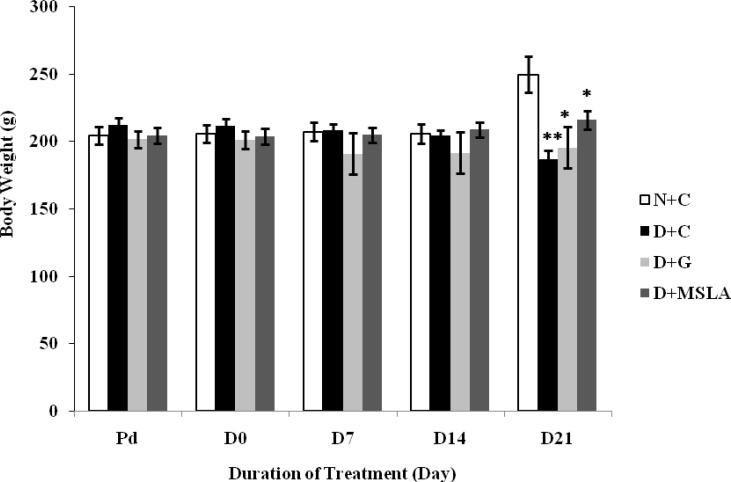
Effect of *M. spicata* on mean body weights (g) of alloxan induced diabetic rats. ^*^P < 0.05, ^**^P < 0.001 Diabetic control rats and diabetic treated rats with Glibenclamide and MSLA were compared with normal control rats

Figure 1 shows the changes in body weight of normal diabetic and on treatment for diabetes rats with glibenclamide and MSLA. Alloxan–induced diabetic rats developed a significant (p˂0.001) decrease in body weight during the 21 days of this experiment. At the end of 21 day treatment, glibenclamide (2mg/kg) and MSLA (300 mg/kg) significantly caused (p˂0.05) increases in body weight**.**

*Effect of MSLA on lipid profile in alloxan-induced diabetic rats*

The results of lipid profile in control and experimental rats treated with glibenclamide and MSLA are shown in Table 3. The level of total cholesterol, triglyceride, and LDL-cholesterol in alloxan-induced diabetic control rats was significantly (p˂0.0001) higher than the normal control. In addition, the level of HDL-cholesterol was reduced in diabetic control rats in comparison to normal control. Oral administration of MSLA (300 mg/kg) developed significant (p˂0.0001) reduction of total cholesterol, triglyceride and LDL-cholesterol levels in treated diabetic rats as compared to diabetic control rats. This result was comparable to the result of the standard drug glibenclamide (2 mg/kg).

*Effect of MSLA on lipid peroxidation in alloxan-induced diabetic rats*

**Figure 2:**
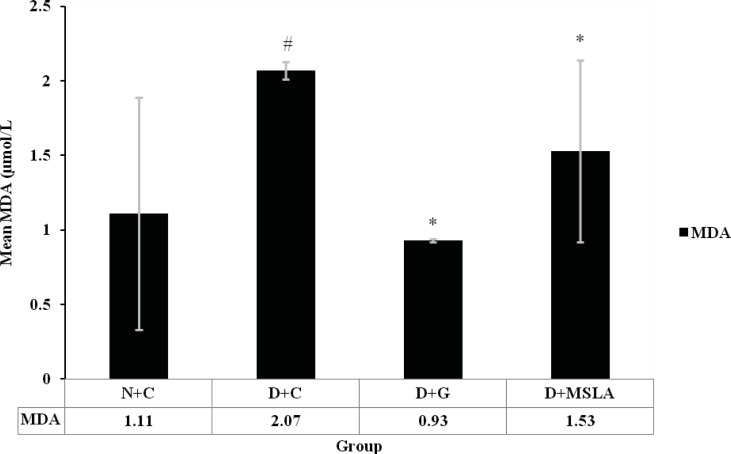
Effect of *M. spicata* on serum MDA level of alloxan induced diabetic rats. ^#^*P*<0.001 Diabetic control rats were compared with normal control rats. ^*^P<0.001 Diabetic treated rats were compared with diabetic control rats

The results of the effect of MSLA on serum MDA *(in vivo* antioxidant activity) in alloxan-induced diabetic rats are depicated in Figure 2. Oral administration of glibenclamide (2 mg/kg) and MSLA (300 mg/kg) caused a significan (p˂0.0001) reduction in the level of MDA compared to diabetic control.

## Discussion

Nowadays various plants, despite the presence of synthetic drugs commonly prescribed to treat diabetes, traditionally are used as antidiabetic agents. Some studies have proven that antidiabetic properties of these plants are related to compounds such as polysaccharides, flavonoids, terpenoids, tannins, and steroids (18). MS leaf aqueous extract, in addition to mentioned constituents, also contains monoterpenoids such as carvone, limonene, menthone, menthol, pulegone, dihydrocarvone and *s*-carveol in spearmint oil (6). Hypoglycemia activity identified in leaves of the plant can be considered as the combined action of these compounds in the leaf extract.

One of the most common substances used for an experimental induction of diabetes mellitus other than streptozotocin, is alloxan monohydrate. In laboratory animals, it induces diaetes by destruction of pancreatic beta cells. As a result of insulin secretion is reduced and consequently increased the level of blood glocuse, cholesterol and triglycerides and decreased body weight (19). 

The results of this study indicate that *Mentha spicata* as a species with high antioxidant activity reduces blood glocuse levels in diabetic rats with alloxan (150 mg/kg). The leaf aqueous extract of *Mentha spicata* plant caused a significnt (p˂0.001) reduction in blood glocuse levels (Table 2). Antidiabetic properties of *Mentha spicata* can be caused by the control of oxidative stress (prevention of free radical formation) induced by alloxan. Oral administration of the leaves of this plant increased activities of serum non-enzymatic antioxidants, erythrocyte antioxidant enzymes and markedly decreased lipid peroxidation in erythrocytes and plasma in diabetic patients (20). Marked increases in serum cholesterol, triglycerides and LDL cholesterol levels are considered as predisposing factors of cardiovascular diseases in diabetes (3). Results of this study clearly indicated elevated serum total cholesterol, triglycerides, and LDL cholesterol in diabetic rats (Table 2). The aqueous extract of *M. spicata* (300 mg/kg) not only reduced the blood glocuse, but also decreased lipid profile. These results were entirely consistent to the results of Barbalho *et al*, (21). Body weight loss is one of the features of diabetes mellitus and for monitoring of severity or response to treatment. Weight measurement is an important tool in the study of diabetes. *M. spicata* leaf aqueous extract at the dose level of 300 mg/kg significantly improved the body weight of diabetic rats (Figure 1). The ability of *Mentha spicata* extract to reduce hyperglycemia may lead to improvement of body weight in diabetic rats (22). 

The previous studies have demonstrated that alloxan-induced diabetes inhibits actions of antioxidant enzymes consequently leading to enhanced production of reactive oxygen species and therefore to increased lipid peroxidation (23, 24). The results of this research showed that formation of MDA as an indicator of lipid peroxidation significantly increased in diabetic rats (Figure 2). The leaf aqueous extract of *Mentha spicata* plant possess the potential of reduction of serum MDA level in rats treated for 21 days. Chadhury *et al*, (5) have proven that the active organic compounds (such as carveol) in complex form with essential transition elements in mint leaves are likely to be responsible for the antioxidant activity of the extract.

## Conclusion

In conclusion, these results clearly demonstrate that *M. spicata* leaf aqueous extract is rich in phenolic compound and has high reducing power. *M spicata*, which has effective hypoglycemia, hypolipidemia and lipid peroxidation activities in diabetic rats, may be useful for the clinical treatment of diabetes. However, studies are on-going for the isolation and purification of bioactive compounds present in the plant extract and for elucidation of its molecular mechanisms

## References

[1] Kim SH, Hyun SH, Choung SY (2006). Anti-diabetic effect of cinnamon extract on blood glucose in db/db mice. J. Ethnopharmacol.

[2] Fowler MJ (2008). Microvascular and macrovascular complications of diabetes. Clin. Diabetes.

[3] Ezeja MI, Anaga AO, Asuzu IU (2015). Antidiabetic, antilipidemic, and antioxidant activities of Gouania longipetala methanol leaf extract in alloxan-induced diabetic rats. Pharm. Biol.

[4] Ahmad W, Khan I, Khan MA, Ahmad M, Subhan F, Karim N (2014). Evaluation of antidiabetic and antihyperlipidemic activity of Artemisia indica linn (aeriel parts) in streptozotocin induced diabetic rats. J. Ethnopharmacol.

[5] Choudhury RP, Kumar A, Garg AN (2006). Analysis of Indian mint (Mentha spicata) for essential, trace and toxic elements and its antioxidant behaviour. J. Pharm. Biomed.Anal.

[6] Arumugam P, Priya NG, Subathra M, Ramesh A (2008). Anti-inflammatory activity of four solvent fractions of ethanol extract of Mentha spicata L investigated on acute and chronic inflammation induced rats. Environ. Toxicol. Pharmacol.

[7] Akdogan M, Kilinç I, Oncu M, Karaoz E, Delibas N (2003). Investigation of biochemical and histopathological effects of Mentha piperita L and Mentha spicata L on kidney tissue in rats. Hum. Exp. Toxicol.

[8] Adelpoor MJ, Golparvar AR (2013). Chemical composition of essential oils of three ecotypes of Mentha spicata L from Kohgiluyeh va Boyer-Ahmad Province, Iran. Iran.J. Herb Drugs.

[9] Kumar A, Chattopadhyay S (2007). DNA damage protecting activity and antioxidant potential of pudina extract. Food Chem.

[10] Zheng J, Wu L-J, Zheng L, Wu B, Song AH (2003). Two new monoterpenoid glycosides from Mentha spicata L. J. Asian Nat. Prod. Res.

[11] Hajjaj Yousuf PM, Yousuf Noba N, Shohel M, Bhattacherjee R, Kumas Das B (2013). Analgesic, anti-inflammatory and antipyretic effect of Mentha spicata (spearmint). Br. J. Pharm. Res.

[12] Moosavy M-H, Esmaeili S, Mostafavi E (2013). Antibacterial effect of Mentha spicata essential oil on Listeria monocytogenes in traditional Lighvan cheese. J. Food Saf.

[13] Kamkar A, Javan AJ, Asadi F, Kamalinejad M (2010). The antioxidative effect of Iranian Mentha pulegium extracts and essential oil in sunflower oil. Food Chem. Toxicol.

[14] Barros L, Ferreira M-J, Queirós B, Ferreira ICFR, Baptista P (2007). Total phenols, ascorbic acid, β-carotene and lycopene in Portuguese wild edible mushrooms and their antioxidant activities. Food Chem.

[15] Yen GC, Duh P Der (1994). Scavenging effect of methanolic extracts of peanut hulls on free-radical and active-oxygen species. J. Agric. Food Chem.

[16] Ashraf H, Heidari RNV (2014). Antihyperglycemic and antihyperlipidemic effects of fruit aqueous extract of Berberis integerrima Bge in streptozotocin-induced diabetic rats. Iran. J. Pharm. Res.

[17] Atawodi SE (2001). Evaluation of the Hypoglycemic, hypolipidemic and antioxidant effects of methanolic extract of “ata-ofa” polyherbal tea (apolyherbal) in alloxan-induced diabetic rats. Drug Invent. Today.

[18] Hooda MS, Pal R, Bhandari A, Singh J (2014). Antihyperglycemic and antihyperlipidemic effects of Salvadora persica in streptozotocin-induced diabetic rats. Pharm Biol.

[19] Rask-Madsen C, King GL (2013). Vascular complications of diabetes: Mechanisms of injury and protective factors. Cell Metabolism.

[20] Ullagaddi R, Mahendrakar P, Bondada A (1999). Efficacy of mint (Mentha spicata L) leaves in combating oxidative stress in type 2 diabetes. Life Sci.

[21] Barbalho SM, Spada APM, Oliveira EP de, Paiva-Filho ME, Martuchi KA, Leite NC, Deus RM, Sasaki V, Braganti LS, Oshiiwa M (2009). Mentha piperita effects on wistar rats plasma lipids. Braz. Arch. Biol. Technol.

[22] Pillai KK, Chidambaranathan N, Halith MM, Narayanan N (2012). Anti-hyperglycemic effect of alcoholic extracts of Cnidoscolus chayamansa in experimental diabetes and their effects on key metabolic enzymes involved in carbohydrate metabolism. Int. J. Res. Pharm. Chem.

[23] Kaleem M, Sheema SH, Sarmad H, Bano B (2005). Protective effects of Piper nigrum and Vinca rosea in alloxan induced diabetic rats. Indian J. Physiol. Pharmacol.

[24] Vincent AM, Russell JW, Low P, Feldman EL (2004). Oxidative stress in the pathogenesis of diabetic neuropathy. Endocrine Rev.

